# The impact of COVID-19 pandemic on inpatient admissions for bipolar disorder

**DOI:** 10.1192/j.eurpsy.2023.1648

**Published:** 2023-07-19

**Authors:** C. Portela, C. Oliveira, D. Areias, M. Gonçalves

**Affiliations:** Hospital de Magalhães Lemos, Porto, Portugal

## Abstract

**Introduction:**

In 2019, there were 39.5 million patients suffering from bipolar disorder worldwide, resulting in around 8.5 million disability-adjusted life years (DALYs) and in a significant economic burden. Bipolar disorder is known to be susceptible to factors that disrupt biological and social rhythms. The COVID-19 pandemic and the measures taken to control it, such as social distancing, home confinement and lockdowns, pose a risk to the stability of bipolar patients. Other factors, for example, reduced access to treatment and stress associated with the disease could also contribute to relapses. Studies have shown that, in 2020, more people with bipolar disorder were hospitalised compared to previous years, including patients without previous history of hospitalizations.

**Objectives:**

This study aims to assess the impact of the COVID-19 pandemic on inpatient admissions for bipolar disorder.

**Methods:**

Socio-demographic and clinical data were collected from electronic medical records. A retrospective observational study of patients who were admitted to a psychiatric ward between March 2019 and February 2021 was conducted. The characteristics of patients admitted before the pandemic (March 2019 to February 2020) and after (March 2020 to February 2021) were compared statistically.

**Results:**

A total of 850 patient admissions were obtained, 15% of which had a main diagnosis of bipolar disorder. The authors will analyze all the variables in the population admitted. The authors expect to find differences between patients hospitalized before and after the beginning of the pandemic, both in clinical presentation (manic, depressive or mixed episode), psychiatric history, comorbidities, suicide attempts and socio-demographic factors.

**Conclusions:**

The COVID-19 pandemic had a significant impact on mental health on a global level, plenty of which is still unknown. The findings of this study will likely show the effects of this crisis on bipolar disorder patients.

**Disclosure of Interest:**

None Declared

